# Hybrid Genome Assemblies of Marine Bacteria Isolated from the Sponge Sycon capricorn

**DOI:** 10.1128/MRA.00858-21

**Published:** 2021-10-28

**Authors:** Oliver L. Mead, Erin E. Hahn, Maja A. Adamska

**Affiliations:** a National Research Collections Australia, Commonwealth Scientific and Industrial Research Organization, Canberra, Australia; b ARC Centre of Excellence for Coral Reef Studies, Research School of Biology, The Australian National University, Canberra, Australia; Montana State University

## Abstract

Sponges have complex relationships with bacteria, the roles of which include food, important components of the holobiont, pathogens, and accidentally accumulated elements of the environment. Consequently, sponges are reservoirs of microbial genomes and novel compounds. Therefore, we isolated and sequenced the whole genomes of bacterial species from the calcareous sponge Sycon capricorn.

## ANNOUNCEMENT

Sponges are filter-feeding, aquatic animals that attach to marine and freshwater substrates. These animals accumulate bacteria that are either digested, released into the environment, or integrated into the microbial communities composing the sponge holobiont (1). Consequently, sponges are reservoirs of microbial genomes and, potentially, novel compounds (2, 3). Therefore, we isolated and cultured bacteria from the calcareous sponge Sycon capricorn, collected from the South-East Australian coast. We sequenced the whole genomes of eight selected strains using short- and long-read technology. The sponges were collected under the New South Wales Parks and Wildlife collection permit numbers P16/0121-1.0 and OUT16/34729.

We collected *S. capricorn* from coastal rocks at approximately three meters’ depth offshore from Orion Beach, New South Wales (NSW), Australia (GPS coordinates, −35.068935, 150.681062) in July 2018. We introduced the sponges into a 16°C laboratory aquarium at the Australian National University (ANU), Canberra, containing previously collected sponges, crustaceans, mollusks, and algae from Jervis Bay. We refreshed the aquarium weekly with 20 liters seawater from Bateman’s Bay, NSW.

We rinsed four *S. capricorn* specimens with sterile artificial seawater (SAS), macerated them using a micropestle, and serially diluted the resulting paste in SAS. We inoculated lysogeny broth plates made with SAS and glucose (LBsg) (peptone [10 g/liter]; yeast extract [5 g/liter]; glucose [10 g/liter]; agar [20 g/liter]) with 100μl of each dilution, then incubated the plates at room temperature (RT) in darkness for 2 weeks. As colonies emerged, we restreaked them onto LBsg, then transferred single colonies of each morphology to 10-ml aliquots of liquid LBsg, and incubated them with shaking at 200 rpm at RT. After 14 days, we isolated DNA from cultures using a protocol optimized for long-read sequencing (4) and long fragment recovery (>3 kb) (5). Using these protocols, shearing is avoided and size selection conducted with solid-phase reversible immobilization (SPRI) beads. These DNA samples were used for the subsequent PCR, short-read, and long-read sequencing reactions.

We amplified the V3 to V4 region of the 16S rRNA gene (6). The Biomolecular Resource Facility (BRF) at ANU Sanger sequenced these amplicons. Comparing the amplicon sequences with the NCBI database using the BLAST server, we identified the bacterial strains to the genus level. We selected eight strains for whole-genome sequencing: 2 *Pseudoalteromonas* spp., 2 *Vibrio* spp., *Francisella* sp., *Shewanella* sp., Neptunomonas phycophila, and a single strain of unknown genus related to *Cobetia*. We prepared short-read sequencing libraries using the Illumina Nextera XT kit and sequenced the pooled libraries on the Illumina MiSeq platform at the BRF. We prepared long-read sequencing libraries using the rapid barcoding sequencing kit (SQK-RBK004; Oxford Nanopore Technologies [ONT], UK) and sequenced the pooled libraries for 16 h on two ONT MinION R9.4 flow cells.

We verified the Illumina read quality using FastQC v.0.11.8 (7) and removed adapters and trimmed reads using Trimmomatic v.0.36 (8), generating 33.2 million paired-end 150-bp reads (Table 1). We base called the long-read data using Oxford Nanopore Guppy v.2.1.3 (9), eliminated reads with Q scores below 7, and demultiplexed the quality-controlled, pooled reads using Guppy_bcsplit.py (10). We removed long-read adapter sequences and split chimeric reads with Porechop v.0.2.4 (11), then filtered the reads using NanoFilt v.2.5.0 (12) (Q score, >8; length, >1,000 bp), generating 4.7 Gb of data. We used Unicycler v.0.4.7 (13) in default hybrid mode to assemble complete circular, or near-complete, genome sequences. We assessed the assembly quality using QUAST v.4.3 (14) and Bandage v.0.8.1 (15) (Table 1). Finally, the genomes were annotated using the NCBI Prokaryotic Genome Annotation Pipeline (PGAP) (16).

**TABLE 1:** Summary of Illumina and Nanopore reads and genome assemblies

Species (isolate no.)	Sample ID	Data for Illumina reads				Data for Nanopore MinION reads				Data for assemblies			
		Total no. of raw read pairs	Total no. of filtered read pairs	Coverage (×)	Total no. of raw reads	Total no. of filtered reads	Mean filtered read length (kbp)	Coverage (×)	BioSample accession no.	*N*_50_ (bp)	No. of contigs >200 bp	Assembled genome size (Mb)	GC content (%)
*Francisella* sp.	Scap27	2,257,467	1,859,017	279	76,622	49,810	10.1	256	SAMN12071515	1,967,290	1 circular	1.97	32.8
Neptunomonas phycophila	Scap9	1,736,335	1,322,630	97	144,771	70,561	8.4	150	SAMN12071509	3,976,365	1 linear	3.98	45.5
*Pseudoalteromonas* sp. (2)	Scap6	2,721,845	2,246,661	160	62,284	39,158	10	95	SAMN12071507	3,381,174	2 circular	4.13	40
*Pseudoalteromonas* sp. (3)	Scap25	2,700,976	2,116,343	149	58,719	36,080	9.6	103	SAMN12071514	3,380,429	2 circular	3.38	40
*Shewanella* sp.	Scap7	2,140,661	1,704,313	98	84,954	51,488	8.9	90	SAMN12071508	5,129,640	1 circular	5.12	45.3
*Vibrio* sp. (2)	Scap16	1,775,232	1,371,440	72	108,398	47,466	9	77	SAMN12071511	1,823,474	15 linear	5.58	44.6
*Vibrio* sp. (3)	Scap24	1,413,849	1,048,807	56	136,881	61,930	9	102	SAMN12071513	3,485,889	1 circular, 1 linear	5.44	44.6
Unknown	Scap17	1,348,043	1,049,698	73	50,421	31,694	8.3	64	SAMN12071512	3,993,610	1 circular, 5 linear	4.13	62.3

### Data availability.

The hybrid genome sequences are available in GenBank under BioProject accession number PRJNA549111. The raw long and short reads can be found under SRA accession number PRJNA549111.
